# Supplementary material for: Cholesterol and SREBP2 Dynamics During Spermatogenesis Stages in Rabbits: Effects of High-Fat Diet and Protective Role of Extra Virgin Olive Oil
Source: Int J Mol Sci. 2025 Apr 25;26(9):4062. doi: 10.3390/ijms26094062 (PMC12071441; doi:10.3390/ijms26094062)
Supplement: Supplementary file 1 [file ijms-26-04062-s001.zip › supplementary material S1.pdf]

Table S1. General components.

**Chemical**	**%**	**Methods**
Humidity and volatile materials	0.06	IRAM 500
Fiber	< 0.01	AOAC
Total Carbon hydrates	< 0.01	Total sugars method
Proteins	< 0.01	Kjeldahl methods, N x 5,8
Calories	899	Kcal /100 g
	3767	Kjoul /100 g
Total lipids	99.94	Twisselmann method

TableS1: Specific chemical components found in EVOO were expressed as a percentage of the analyzed material. IRAM: international standards (www.iram.org.ar, Instituto Argentino de Normalización y Certificación = Normalization and certification Argentinian institute).

Table S2. EVOO fatty acid chromatography.

**Class**	**Percentage of methyl esters**
Palmitic	14.25 ± 0.24
Palmitoleic	1.48 ± 0.01
Heptadecanoic	0.07 ± 0.01
Stearic	2.07 ± 0.08
Oleic	70.36 ± 0.28
Linoleic	9,92 ± 0.70
Linolenic	0.72 ± 0.26
Arachidonic	0.37 ± 0.10
Behenic	0.11 ± 0.01

Table S2: The fatty acid composition was expressed as a percentage of methyl esters ± SD.

Table S3. Determination of acidity and peroxide index of olive oil.

**Acidity: g% % oleic acid**	0,79±0.1
**Peroxide index: meq O_2/kg**	9.0±0.5

Table S3: Acidity (ISO standard 660 – 1996 / amd 1: 2003) was expressed as a percentage and peroxide index (ISO standard: 3960:2007) as meq.
