# Supplementary material for: Cholesterol and SREBP2 Dynamics During Spermatogenesis Stages in Rabbits: Effects of High-Fat Diet and Protective Role of Extra Virgin Olive Oil
Source: Int J Mol Sci. 2025 Apr 25;26(9):4062. doi: 10.3390/ijms26094062 (PMC12071441; doi:10.3390/ijms26094062)
Supplement: Supplementary file 1 [file ijms-26-04062-s001.zip › supplementary material S2.pdf]

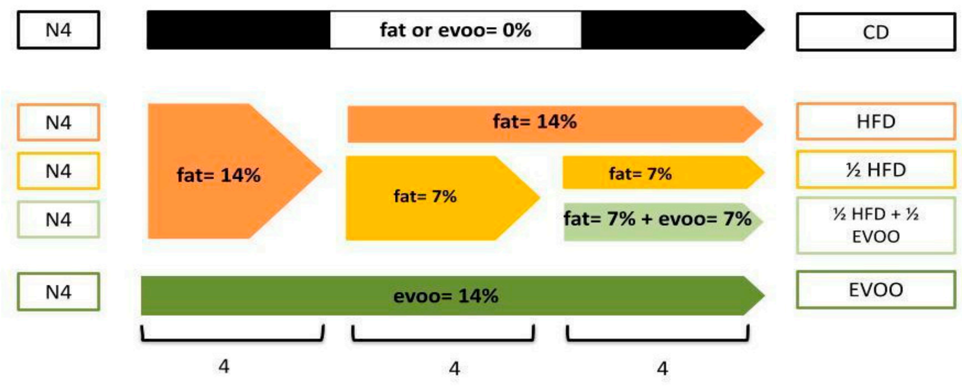

Figure S1. Experimental design. The number of animals (N), cow grease (fat), olive oil (evoo), or a combination of both (v/w) added to the rabbit commercial diet, and the group name used in experimental design. All rows correspond to 4 rabbits feeding during 12 months (CD = black arrow - first row, HFD = orange arrows - second rows, and EVOO = dark green arrow - row five), or combination of diets for at least 4 months, ½ HFD begin with 14% fat (orange arrow) and then received 4 + 4 months of 7% of fat (½ HFD, yellow arrows - third rows), but to promote protection the last 4 months received 7 %.
