# Supplementary material for: Cholesterol and SREBP2 Dynamics During Spermatogenesis Stages in Rabbits: Effects of High-Fat Diet and Protective Role of Extra Virgin Olive Oil
Source: Int J Mol Sci. 2025 Apr 25;26(9):4062. doi: 10.3390/ijms26094062 (PMC12071441; doi:10.3390/ijms26094062)
Supplement: Supplementary file 1 [file ijms-26-04062-s001.zip › supplementary material S3.pdf]

Table S4: Multiple comparisons (Tukey test) corresponding to Filipin staining, according to the zone/stage of the seminiferous tubule epithelium, in each experimental group.

Diet	vs diet	significance	
Z 1			
CD	HFD	****	< 0.0001
CD	½ HFD	*	0.0447
CD	EVOO	ns	0.899
CD	½ HFD + ½ EVOO	**	0.0074
HFD	½ HFD	****	< 0.0001
HFD	EVOO	****	< 0.0001
HFD	½ HFD + ½ EVOO	****	< 0.0001
½ HFD	EVOO	ns	0,5882
½ HFD	½ HFD + ½ EVOO	ns	0,6072
EVOO	½ HFD + ½ EVOO	ns	0.2087
Z2			
CD	HFD	****	< 0.0001
CD	½ HFD	****	< 0.0001
CD	EVOO	ns	0.0943
CD	½ HFD + ½ EVOO	**	0.0098
HFD	½ HFD	**	0.0049
HFD	EVOO	****	< 0.0001
HFD	½ HFD + ½ EVOO	***	0.0003
½ HFD	EVOO	**	0.013
½ HFD	½ HFD + ½ EVOO	ns	0.4978
EVOO	½ HFD + ½ EVOO	ns	0.2272
Z3			
CD	HFD	***	0.0001
CD	½ HFD	***	0.0009
CD	EVOO	ns	0.9902
CD	½ HFD + ½ EVOO	ns	0.6852
HFD	½ HFD	ns	0.1475
HFD	EVOO	****	< 0.0001
HFD	½ HFD + ½ EVOO	***	0.0006
½ HFD	EVOO	****	< 0.0001
½ HFD	½ HFD + ½ EVOO	***	0.0003
EVOO	½ HFD + ½ EVOO	ns	0.173
